# Photocatalytic Degradation of Atrazine under Visible Light Using Novel Ag@Mg_4_Ta_2_O_9_ Nanocomposites

**DOI:** 10.1038/s41598-019-43915-y

**Published:** 2019-05-16

**Authors:** Nazeeha S. Alkayal, Mahmoud A. Hussein

**Affiliations:** 1 Chemistry Department, Faculty of Science, King Abdulaziz University, P.O. Box 80203, Jeddah, 21589 Saudi Arabia; 2 Chemistry Department, Faculty of Science, Assiut University, Assiut, 71516 Egypt

**Keywords:** Nanoparticles, Photocatalysis

## Abstract

In this work, a novel as well as an efficient photocatalyst based Ag@Mg_4_Ta_2_O_9_ nanoparticles have been prepared for the photocatalytic degradation of atrazine using the hydrothermal technique. In order to measure the chemical composition as well as the phase of the novel nanoparticles, different characterization techniques were applied to confirm their structures. Furthermore, the percent of Ag in the Ag@Mg_4_Ta_2_O_9_ nanoparticles has been investigated on the properties of Mg_4_Ta_2_O_9_ (physical and chemical). The phase of new Ag@Mg_4_Ta_2_O_9_ was confirmed via XRD data comparing with pure Mg_4_Ta_2_O_9_ phase. The images of the morphologies for all samples were studied using TEM with pore size distribution around 24 nm for 2.0 wt.% Ag@Mg_4_Ta_2_O_9_ nanocomposite. The new Ag@Mg_4_Ta_2_O_9_ nanoparticles have been applied for atrazine degradation using photocatalytic method. Due to the high BET surface area and low band gap, the nanoparticles with 2.0 wt.% of Ag@Mg_4_Ta_2_O_9_ display the best photocatalyst efficiency for atrazine degradation. Moreover, the application and the limitation of the photodegradation process were estimated. Different conditions effect on atrazine degradation such as dosages of photocatalyst and concentration of atrazine were investigated. The ability of 2.0 wt.% Ag@Mg_4_Ta_2_O_9_ for atrazine degradation was reused for many times with the same efficiency.

## Introduction

A large number of materials of different origins have been found in ground water, surface, sewage and even drinking water as a significant class of organic pollutants in the last few decades. The biggest concern is their adverse health effects for the human and environment^1–4^. The outmost critical health issue include in most of countries in the world putting ashore of wastewater coming mainly form industrial sources^5^. Pesticides are one of the most essential categories of organic pollutants. Most of organic pollutants including pesticides, which have been displayed in water, are coming from many different resources taking in our consideration wastewater sources. As reported, almost all types of pesticides show a strong environmental impendence on the world inhabitance. In addition, all types of pesticides show forthright direct influence on the human health as well as show extremely high toxic activity^6,7^. Bactericides, herbicides, insecticides fungicides and many other derivatives are all classified as different types of pesticides^8^. Furthermore, atrazine, chlorothalonil, methamidophos, chlorpyrifos, cypermethrin, etc. have been also classified as ordinary and famous pesticides too^9,10^. The literature says, all types of underground and/or surface water including: tape and fresh water display significant pesticides contaminations^11–13^. It is also reported that, tape and fresh water sources with concentrations (500 ppm) display higher contamination of different types of pesticides^14^. A lot of techniques were reported for wastewater treatments as favorable techniques. These methods include membrane filtration, adsorption, oxidation & biosorption and photocatalytic degradation^15–19^. Photocatalytic degradation technique is considered as the most favorable method which has been utilized in wastewater remediation from variable sources. The good photocatalyst should have enhanced photocatalytic activity, facile regeneration producibility, and higher photocatalytic stability^20,21^. On the other hand, the new hot branch of science that deals with layout, preparation, and application of small elements and/or molecules in nm range is called nanotechnology^22–24^. Such small tiny compounds and/or elements show a lot of amazing properties and advantages which help them to be utilized for variable applications. Huge number of nanomaterials of different types have been formerly reported as excellent photocatalysts^25–29^. A special attention has been recently given to the ultra-small Au clusters and /or graphene supported nanocomposites for enhanced photocatalytic & photoredox catalysis behavior^30–32^. The most widely used nanomaterials is titanium dioxide (TiO_2_) which has been used for the photocatalytic degradation of a lot of organic pollutants. A special attention has been reported for the photocaltalytic degradation of pesticides using TiO_2_ nanoparticles^33–35^. This is mainly is due to its low price, commercial availability, very low toxicity, higher photocatalytic activity, higher thermal stability, as well as sensible photochemical stability^25–28^. TiO_2_ is also classified as an efficient distinct photocatalyst that has been predestined for wastewater treatments. In addition it has a suitable redox behavior comparing to the rest of photocatalysts that have been utilized. Anatase, rutile and brookite represent the major crystal phases for TiO_2_ that have been reported; but formally the anatase phase act as the most efficient as well as efficacious phase^29^. Furthermore, TiO_2_ displaying a higher activity while interact over UV radiation coming from natural or artificial sunlight. Where from the solar spectrum (3% to 5%) has been nominated as a results of its expansive band gab (E_g_ = 3.2 eV). For that purpose, the scientific workers doing their best to develop a new design of efficient nanomaterials to effectively minimize its band gab^18^.

On the other hand, the form Mg_4_Ta_2_O_9_ is considered as one of the most important reported three forms of a binary MgO–Ta_2_O_5_ system. From the crystal structure point of view of Mg_4_Ta_2_O_9_, it is easily to detect that, Mg_4_Ta_2_O_9_ display a corundum structure as a common crystallization form which can be derived from a hexagonal close packing of oxygen atoms with two-thirds of the octahedral sites occupied by Mg and Ta atoms; rather than, the other forms display a trirutile structure^36–40^. The study of Mg_4_Ta_2_O_9_ nanomaterials shows a considerable interest due to its fantastic and powerful properties which leads to its use over a wide range of industrial applications; moreover, Mg_4_Ta_2_O_9_ nano-powder was successfully prepared as reported in the literature by different working groups^41–43^. Therefore, it is important to develop a new design of modern nanomaterials to be applied in the field of photocatalysts degradation. Hence, the current manuscript is aimed to synthesize novel Ag@Mg4Ta2O9 composite materials known through hydrothermal technique. Cyclohexylamine has been consumed for synthetization of Mg_4_Ta_2_O_9_ as a matrix during the operated process. Moreover, the role of Ag loading on the total performance of Mg_4_Ta_2_O_9_ including its physical as well as chemical has been studied through its related nanocomposites formation with a general formula in the form of Ag@Mg_4_Ta_2_O_9_. Furthermore, the photocatalytic degradation of these new fabricated products will be tested against atrazine as an important application of such materials. All the optimization procedures which have a direct effect on degradation of atrazine will be also studied discussed and evaluated in details. Such procedures include: atrazine concentration, photocatalytic performance, amount of used photocatalyst and all other factors.

## Experimental

### Materials

Tantalum isopropoxide, magnesium isopropoxide, cyclohexylamine, silver nitrate and methanol were purchased from Sigma-Aldrich and used as purchased without any extra purification process.

### Ag doped Mg_4_Ta_2_O_9_ nanoparticles preparation

A typical method to synthesize the required Mg_4_Ta_2_O_9_ nanoparticles is summarized in the following few lines. Two separated solutions (1 and 2) were prepared as follows. Solution 1: cyclohexylamine (0.02 g) was dissolved in a mixture of ethanol (40 mL) and double distilled water about (60 mL); after that tantalum isopropoxide (8 mmol) was added. The mixture was stirred for 60 min. However, solution 2: magnesium isopropoxide (16 mmol) was directly mixed with the same mixture of ethanol and double distilled water (40 mL and 30 mL respectively). Then, solution 2 was added to solution 1 and continues stirring for 60 min. The total mixture transferred to a Teflon-lined autoclave at 180 °C for 24 hrs. Then, the whole reaction mixture was gradually cooled to room temperature and the final products were collected. Ethanol, double distilled water, and acetone were used to wash the materials, and then eventually dried at 100 °C for 24 h.

Deposition process used to synthesize Ag@Mg_4_Ta_2_O_9_ samples. An ideal method to prepare the Ag@Mg_4_Ta_2_O_9_ is as follows. Under sonication irradiation, Mg_4_Ta_2_O_9_ nanoparticles were dispersed in 100 mL distilled water. Before irradiation using strong UV lamp, the silver nitrate solution was slowly added to the Mg_4_Ta_2_O_9_ suspension for 24 hrs. Finally, Ag@Mg_4_Ta_2_O_9_ nanoparticles were obtained after drying of the materials for 24 h at 80 °C. The silver contents that loading on Mg_4_Ta_2_O_9_ were determined as 0.5, 1.0, 1.5, 2.0 and 2.5 wt., and denoted as (x wt.%) Ag@Mg_4_Ta_2_O_9_ and x is wt.% of silver.

### Characterization techniques

The normal and popular characterization procedures were adopted to identify the synthesized nanoparticles as follows: X-ray diffraction (XRD) analysis that applied to detect the crystalline phase (using Bruker axis D8 with Cu Kα radiation instrument at RT). Nanostructure and surface morphologies were checked out applying JEOL-JEM-1230 transmission electron microscopy (TEM). To perform this measurement, the specimens were suspended in ethanol and subjected to ultrasonicator for nearly 30 min, after that; a tiny dose of the suspended and sonicated solution was left to dry on a copper grid coated with carbon and loaded into the TEM instrument. A Thermo Scientific spectrometer (K-ALPHA-type) was adopted to perform the X-ray photoelectron spectroscopy (XPS) measurements. Surface characterization was accomplished applying Nitrogen-adsorption assessments on the specimens with a Chromatech instrument (Nova 2000 series). On the other hand, a fluorescence spectrophotometer (Shimadzu RF-5301) was adopted to display the Photoluminescence emission spectra (PL). Whereas, UV-Vis-NIR spectrophotometer (V-570, Jasco, Japan) was supported in order to estimate the band gap performance from the UV-Vis diffuse reflectance spectra (UV-Vis-DRS); the experiment was performed in normal condition (air) at ambient temperature to identify absorption from 200 up to 800 nm.

### Photocatalytic activity

Visible light radiation was utilized as an important tool to determine the catalytic activity of the produced materials and to measure the atrazine degradation as well. 300-W Xenon lamp was utilized as a main origin of irradiation during the running experiments. Together with an optical cut-off filter which is important to cancel other extra illumination below the wavelength of 420 nm. Prior the illumination process, a solution of atrazine (100 ppm) was stored in a murky place for half an hour to insure that a complete equilibrium (adsorption-desorption) was achieved. The changes in the atrazine concentrations during the experiments were obviously determined using A Shimadzu LC 20 A High-pressure liquid chromatography with a C18 column UV detector. Moreover, atrazine photoxidation produces a different tiny particles was clearly examined throughout measuring its concentrations by using DX-300 ion chromatography in the presence of a CDM-II conductivity detector as well as an AS4A-SC column. These particles include: carbon dioxide, NO_3_ and Cl ions. The end material produced by the atrazine photoxidation process include carbon dioxide gas which is confirmed by passing the evolved flow gases meanwhile a sodium hydroxide solution (0.2 M). Barium nitrate solution precipitate a white material as an end material; filtered off, washed, dried and analyzed by common predictable techniques.

## Results and Discussion

In the current research, novel materials of Ag@Mg_4_Ta_2_O_9_ nanocomposites have been synthesized using hydrothermal technique. In addition study the performance of the new synthesized Mg_4_Ta_2_O_9_ nanocomposites in the presence of doped Ag of variable loading including its physical and chemical properties as well. The new nanoparticles were characterized using different techniques such as TEM, BET, XPS, UV-Vis spectra, XRD, PL and N_2_ adsorption-desorption isotherms.

### Photocatalysts identification and characterizations

**Figure 1:**
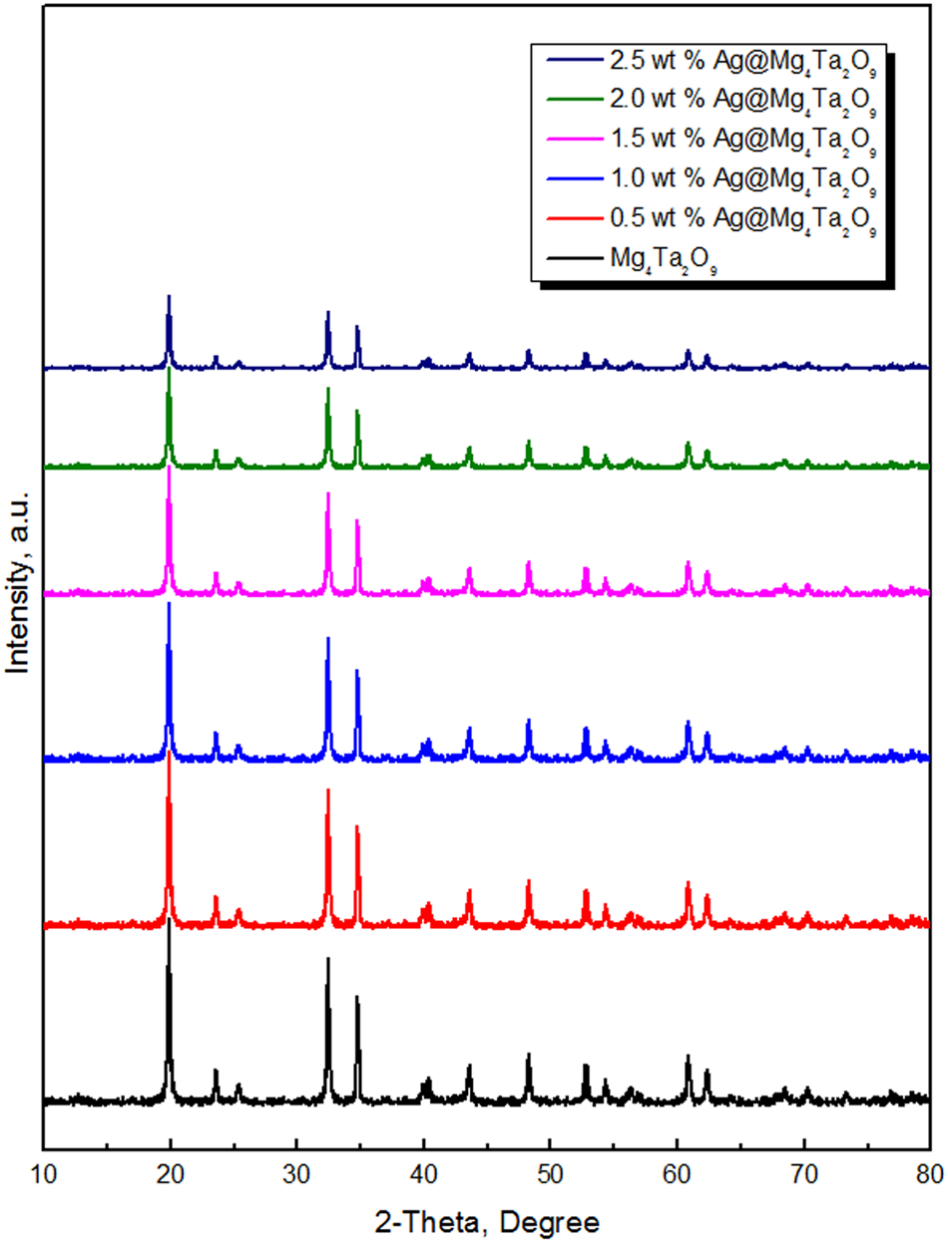
XRD patterns Mg_4_Ta_2_O_9_ and Ag@Mg_4_Ta_2_O_9_ nanocomposites.

The XRD diffractograms of pure Mg_4_Ta_2_O_9_ and new Ag@Mg_4_Ta_2_O_9_ nanoparticles are displayed in Fig. 1. XRD patterns approve that the formation of Ag@ Mg_4_Ta_2_O_9_ nanocomposite in the same phase of pure Mg_4_Ta_2_O_9_. Because of the low loading of silver on Mg_4_Ta_2_O_9_ surface, there is no more bands for Ag or Ag_2_O are detected for new Ag@Mg_4_Ta_2_O_9_ materials. Furthermore, the result shows that the increasing in the percent of silver content leads to significant decrease of the bands of Mg_4_Ta_2_O_9_. In addition, The new Ag@Mg_4_Ta_2_O_9_ nanoparticles for Ag@Mg_4_Ta_2_O_9_, 0.5 wt% Ag@Mg_4_Ta_2_O_9_, 1.0 wt% Ag@Mg_4_Ta_2_O_9_, 1.50 wt% Ag@Mg_4_Ta_2_O_9_, 2.0 wt% Ag@Mg_4_Ta_2_O_9_ and 2.50 wt% Ag@Mg_4_Ta_2_O_9_ have crystallite size values equal 24, 20, 18, 16, 14 and 12 nm respectively. The result shows the size of the obtained Mg_4_Ta_2_O_9_ effect with the silver content.

**Figure 2:**
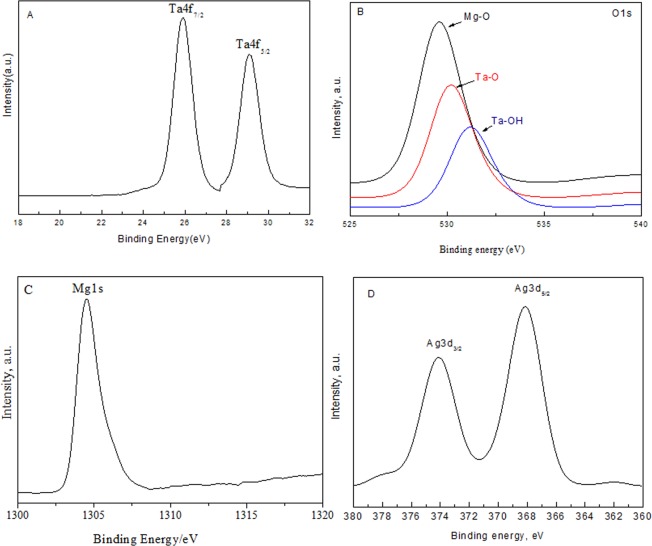
XPS spectra of 2.0 wt% Ag@Mg_4_Ta_2_O_9_ sample, where (**A**) Ta4f; (**B**) O1s; (**C**) Mg1s and (**D**) Ag3d.

According to the existence of different binding energies for Ta 4f 7/2 at 26.2 eV and Ta 4f 7/2 at 29.0 eV, the Ta^5+^ ions are highly present in the XPS spectrum of Ta species in Fig. 2A. Figure 2B shows that the three binding energy peaks for oxygen at 529.6, 530.0 and 531.1 eV that confirm the presence of three form of oxygen, such as Mg-O, Ta-O and Ta-OH, respectively. However, the XPS spectrum of Mg particles in high-resolution is shown in Fig. 2C. The observed data also prove the existence of Mg^2+^ ions which is mainly attributed to the existence of binding energy value at 1304.5 eV which is due to Mg-O. Also, Fig. 2D shows the XPS spectrum of Ag particles with high-resolution. The results signalize that the two binding energies peaks at 368.2 eV and 374.1 eV, are mainly attribute to Ag3d_5/2_ and Ag3d_3/2_ respectively. This observation approves the presence of silver metal as expected.

**Figure 3:**
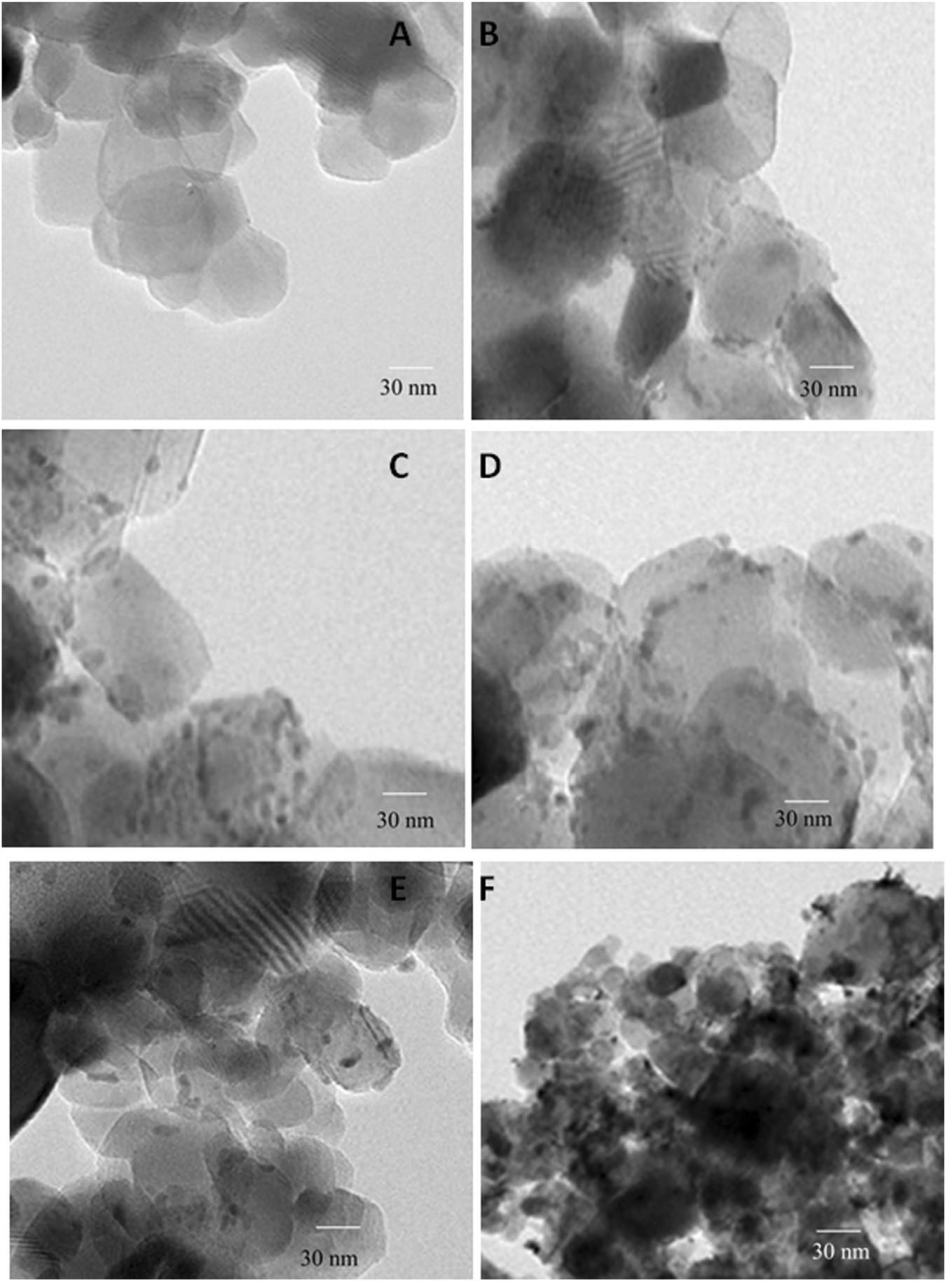
TEM images of Mg_4_Ta_2_O_9_ and Ag@Mg_4_Ta_2_O_9_, where (**A**) Mg_4_Ta_2_O_9_; % (**B**) 0.5 wt% Ag@Mg_4_Ta_2_O_9_; (**C**) 1.0 wt% Ag@Mg_4_Ta_2_O_9_; (**D**) 1.50 wt% Ag@Mg_4_Ta_2_O_9_; (**E**) 2.0 wt% Ag@Mg_4_Ta_2_O_9_ and (**F**) 2.50 wt% Ag@Mg_4_Ta_2_O_9_ samples.

As revealed by the TEM images in Fig. 3, the morphological features for Mg_4_Ta_2_O_9_ and the new Ag@Mg_4_Ta_2_O_9_ nanoparticles showed that the silver was doped on the surface of Mg_4_Ta_2_O_9_ as spots. It is established that the size of Mg_4_Ta_2_O_9_ effect of silver content which come to an agreement with XRD results.

**Figure 4:**
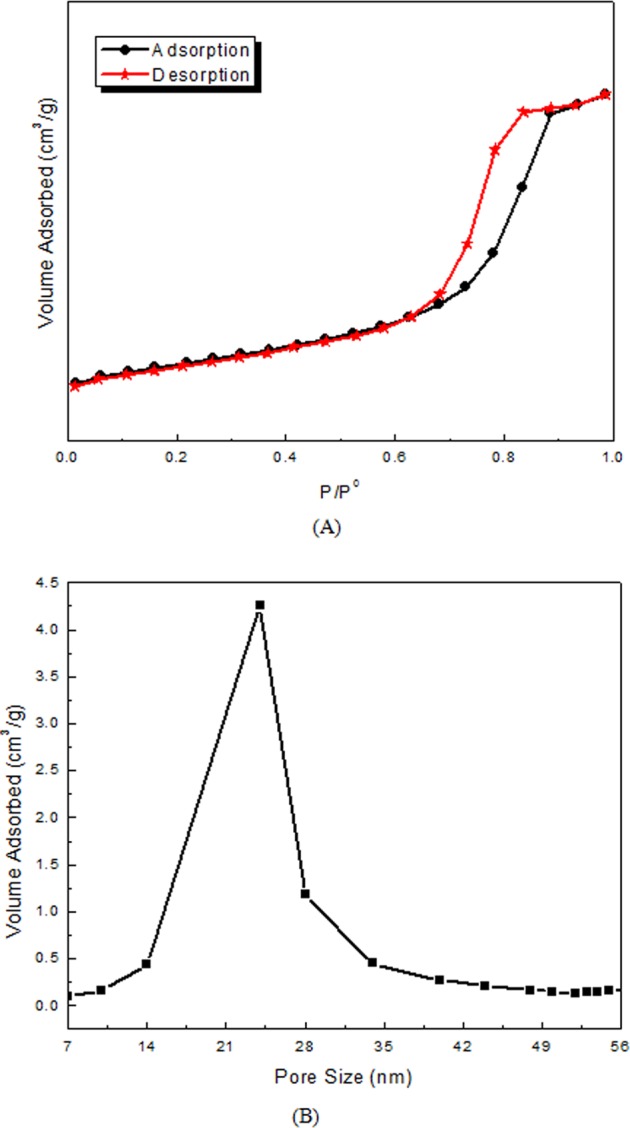
(**A**) N_2_ adsorption-desorption isotherms of 2.0 wt% Ag@Mg_4_Ta_2_O_9_ sample; (**B**) Pore size distribution curve of 2.0 wt% Ag@Mg_4_Ta_2_O_9_ sample.

The porosity of 2.0 wt.% Ag@Mg_4_Ta_2_O_9_ was determined using N_2_ adsorption desorption isotherms and pore size distribution curve (Fig. 4A,B) respectively. Appearance of narrow hysteresis and pore size distribution of 2.0 wt.% Ag@Mg_4_Ta_2_O_9_ indicates the occurrence of mesoporous in the networks. The pore size of 2.0 wt.% Ag@Mg_4_Ta_2_O_9_ is calculated using the BJH method and it has about 24 nm as revealed in Fig. 4B. Table 1 illustrates BET surface area of pure Mg_4_Ta_2_O_9_ and new Ag@Mg_4_Ta_2_O_9_ nanoparticles. The result shows the increasing of silver content leads to decrease BET surface area of Mg_4_Ta_2_O_9_.

**Table 1:** BET surface area and band gap of Mg_4_Ta_2_O_9_ and Ag@Mg_4_Ta_2_O_9_ nanocomposites.

Sample	Band gap energy, eV	BET surface area, m^2^/g
Mg_4_Ta_2_O_9_	3.70	50.0
0.5 wt% Ag@Mg_4_Ta_2_O_9_	3.20	48.0
1.0 wt% Ag@Mg_4_Ta_2_O_9_	3.00	46.0
1.5 wt% Ag@Mg_4_Ta_2_O_9_	2.84	44.0
2.0 wt% Ag@Mg_4_Ta_2_O_9_	2.59	42.0
2.5 wt% Ag@Mg_4_Ta_2_O_9_	2.58	40.0

**Figure 5:**
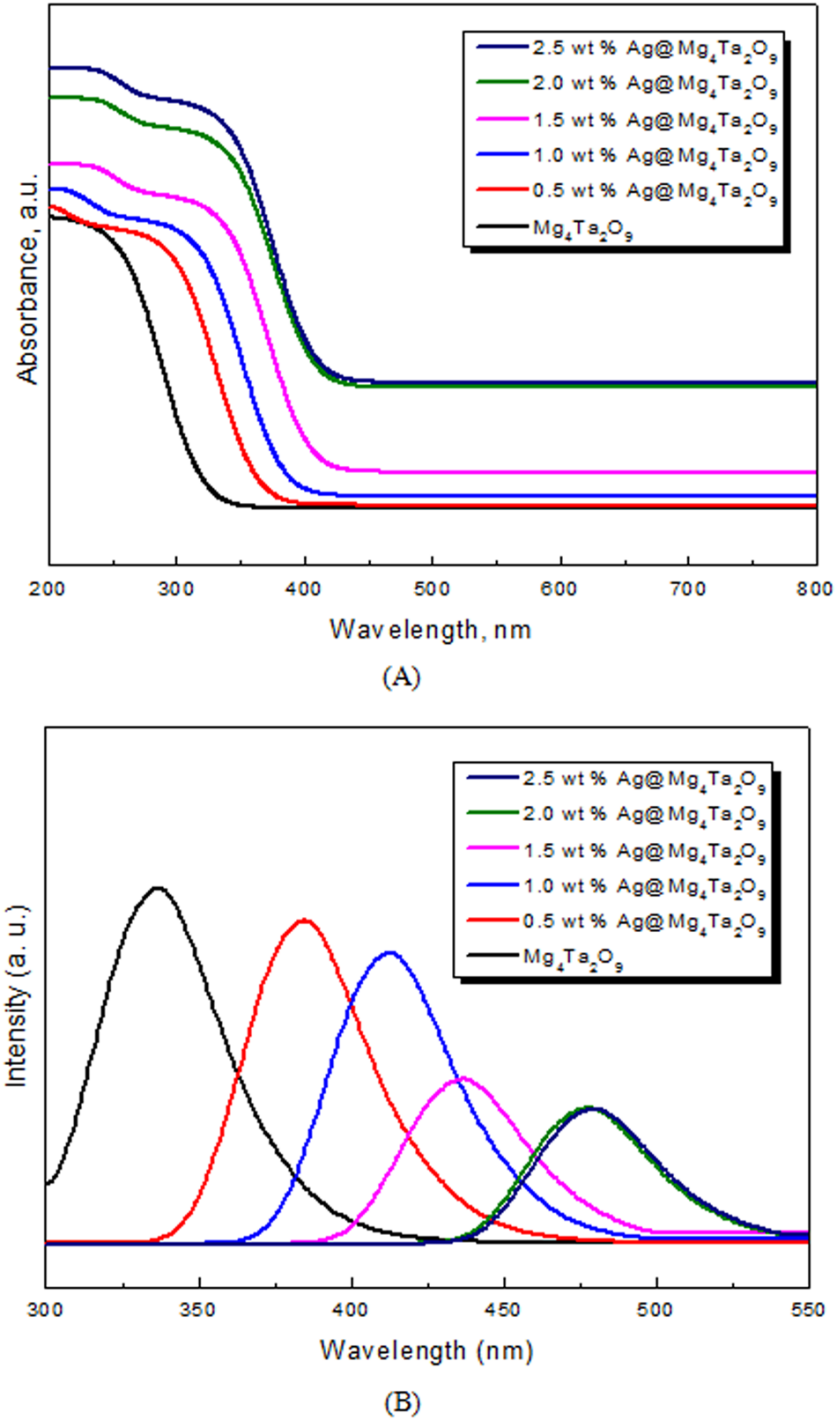
(**A**) UV-Vis spectra of Mg_4_Ta_2_O_9_ and Ag@Mg_4_Ta_2_O_9_ nanocomposites; (**B**) PL spectra of Mg_4_Ta_2_O_9_ and Ag@Mg_4_Ta_2_O_9_ nanocomposites.

Figure 5(A) illustrates the UV-Vis spectra of Mg_4_Ta_2_O_9_ and all Ag@Mg_4_Ta_2_O_9_ nanocomposites samples. The absorption peak of Mg_4_Ta_2_O_9_ is shifted to higher wavelengths going from silver addition. Table 1 also shows the band gap energy of Mg_4_Ta_2_O_9_ and all new Ag@Mg_4_Ta_2_O_9_ samples by calculating from their respective UV-Vis spectra. The values of band gap are 3.7, 3.2, 3.00, 2.84, 2.59 and 2.58 eV for Mg_4_Ta_2_O_9_ and Ag@Mg_4_Ta_2_O_9_, 0.5 wt% Ag@Mg_4_Ta_2_O_9_, 1.0 wt% Ag@Mg_4_Ta_2_O_9_, 1.50 wt% Ag@Mg_4_Ta_2_O_9_, 2.0 wt% Ag@Mg_4_Ta_2_O_9_ and 2.50 wt% Ag@Mg_4_Ta_2_O_9_ samples, respectively. Thus, while the percent composition of silver increased from 0 to 2.0 wt%, the band gap of energy decreased for Mg_4_Ta_2_O_9_ from 3.70 to 2.59 eV, respectively. The data shows that above 2.00 wt% of Ag on Mg_4_Ta_2_O_9_ has no significant effect on the band gap energy of Mg_4_Ta_2_O_9_. As a result, the best wt% of Ag is 2.00 wt%.

Figure 5(B) shows the PL spectra of Mg_4_Ta_2_O_9_ and Ag@Mg_4_Ta_2_O_9_ nanoparticles. The next order displays the decreasing in the intensity peak: pure Mg_4_Ta_2_O_9_ > 0.5 wt% Ag@Mg_4_Ta_2_O_9_ > 1.0 wt% Ag@Mg_4_Ta_2_O_9_ > 1.5 wt% Ag@Mg_4_Ta_2_O_9_ > 2.0 wt% Ag@Mg_4_Ta_2_O_9_ > 2.5 wt% Ag@Mg_4_Ta_2_O_9_. By using the PL emission spectra, the band gap energy values of Mg_4_Ta_2_O_9_ and its corresponding Ag@Mg_4_Ta_2_O_9_ nanoparticles are 0.5 wt% Ag@Mg_4_Ta_2_O_9_, 1.0 wt% Ag@Mg_4_Ta_2_O_9_, 1.50 wt% 3.72, 3.22, 3.02, 2.86, 2.60 and 2.61 eV, respectively with agreement with the UV-Vis data.

### Photocatalytic activities

**Figure 6:**
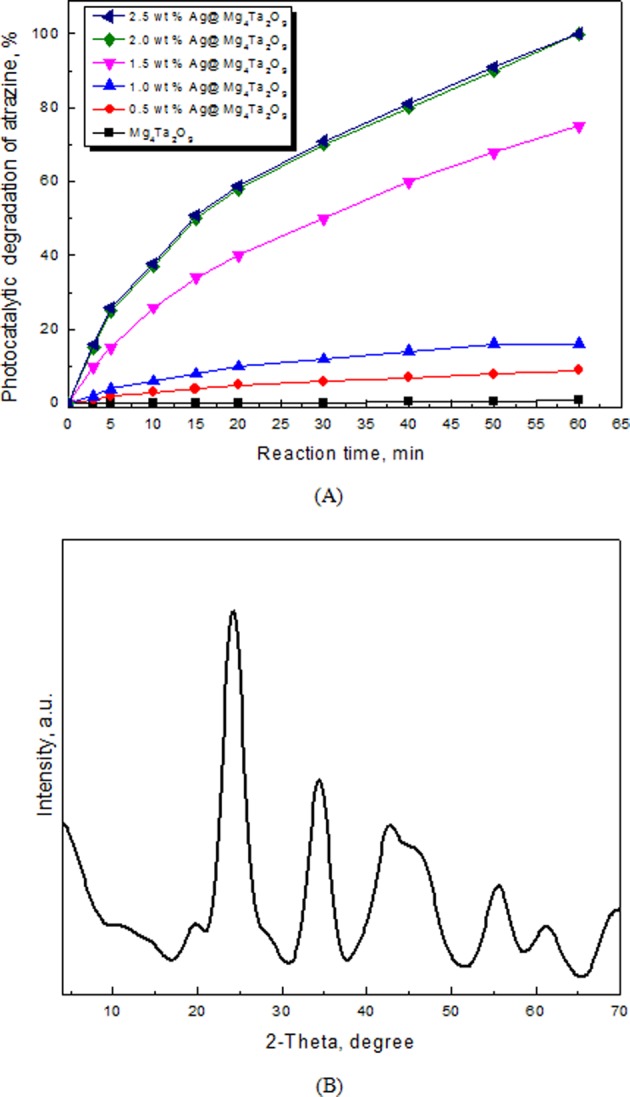
(**A**) Effect of weight percent of Ag on photocatalytic activity of Mg_4_Ta_2_O_9_ nanocomposites for degradation of atrazine; (**B**) XRD patterns of the white precipitate.

Figure 6(A) displays the photocatalytic activity of Ag@Mg_4_Ta_2_O_9_ nanoparticles for atrazine degradation. The result shows the addition of silver weight has significant effect on the photocatalytic activity of Mg_4_Ta_2_O_9_. While the loading of silver percent increased from 0 to 2.0 wt%, the photocatalytic activity enhanced from 1 to 100%. However, above 2.0 wt% of silver shows no effect on photocatalytic activity of Mg_4_Ta_2_O_9_. The data confirms that the composition of silver acts a significant factor for control band gap of Mg_4_Ta_2_O_9_. Therefore, the most Ag@Mg_4_Ta_2_O_9_ nanocomposite displays as effective photocatalyst for atrazine degradation is 2.0 wt% Ag@Mg_4_Ta_2_O_9_ nanoparticles.

The nature of gases that begin through the photocatalyst chemical reaction is investigated. The photocatalytic reaction pumped an outflow throughout a sodium hydroxide solution (0.2 M). A white precipitate obtained, after adding barium nitrate solution. Figure 6(B) shows the XRD pattern for the white precipitate that promise the formation of barium carbonate which consider as the related carbonate salt with a correct card number (05-0378). The data mentions that the most important gas evolved during the atrazine photocatalytic oxidation is carbon dioxide. In the complete photocatalytic oxidation of atrazine, nitrate, H_2_O, carbon dioxide and chloride ions are confirmed as final products during this process using 2.0 wt% Ag@Mg_4_Ta_2_O_9_ nanoparticles.

**Figure 7:**
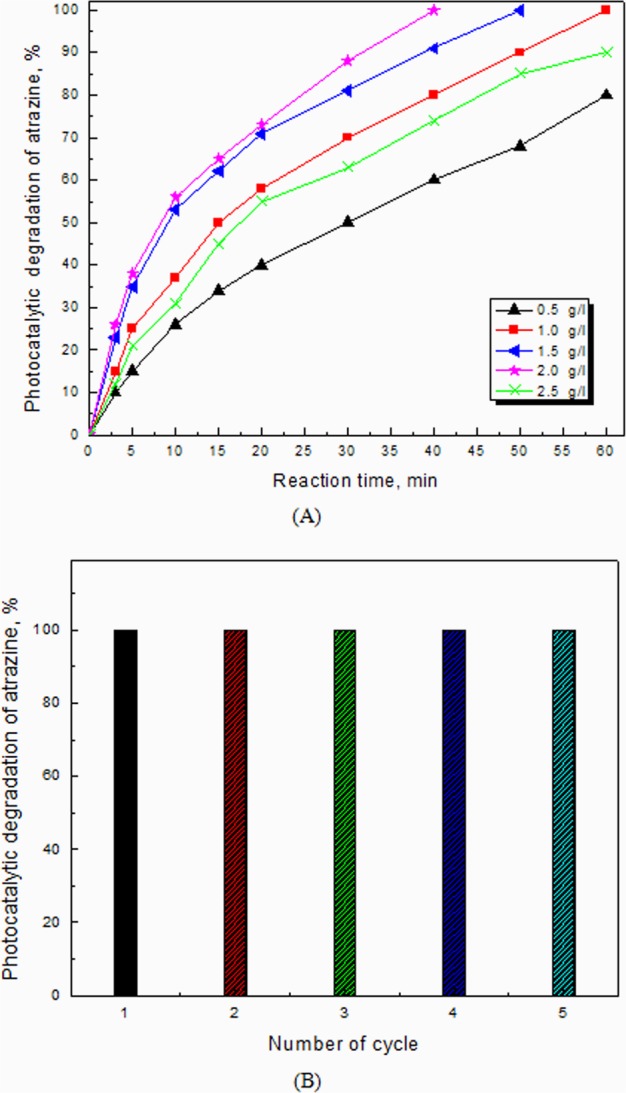
(**A**) Effect of dose of 2.0 wt% Ag@Mg_4_Ta_2_O_9_ photocatalyst on photocatalytic activity of Mg_4_Ta_2_O_9_ nanocomposites for degradation of atrazine; (**B**) Recycling and reuse of 2.0 wt% Ag@Mg_4_Ta_2_O_9_ photocatalyst for degradation of atrazine.

As shown in Fig. 7(A), the effect of 2.0 wt% Ag@Mg_4_Ta_2_O_9_ nanocomposite as a photocatalyst in the degradation of atrazine illustrates the activity of such photocatalyst improved from 80 to 100% by increasing the dosage of 2.0 wt% Ag@Mg_4_Ta_2_O_9_ catalyst within the range from 0.5 to 1.0 g/L, respectively. In order to complete the atrazine decomposition, time required is found to be from 60 to 40 min by increasing the dose of 2.0 wt% Ag@Mg_4_Ta_2_O_9_ nanocomposite within the range from 1.0 to 2.0 g/L, respectively. While the dose of 2.0 wt% Ag@Mg_4_Ta_2_O_9_ increases, the corresponding photocatalytic oxidation of atrazine is regularly increased through increasing the total number of active sites. However and while using an over dose (above 2.0 g/L) from the same photocatalyst 2.0 wt% Ag@Mg_4_Ta_2_O_9_, the activity of photocatalyst is obviously reduced. As a result, 2.0 g/L dose shows the best dose using for photocatalytic oxidation reaction because of after using over 2.0 g/L dose, the light scattering to the photocatalyst surface will be highly forbidden and hindered.

The reusing and recycling of 2.0 wt% Ag@Mg_4_Ta_2_O_9_ nanocomposite as photocatalyst for degradation of atrazine was studied as illustrated in Fig. 7(B). The data shows the efficiency and stability of this photocatalyst against atrazine decomposition is planned for five times.

## Conclusions

A new set of Ag@Mg_4_Ta_2_O_9_ nanocomposites via hydrothermal method technique has been prepared using different composition of silver. Furthermore, the physical and chemical properties of Mg_4_Ta_2_O_9_ have been affected of Ag content in Ag@Mg_4_Ta_2_O_9_ nanocomposites. According to lower loading of Ag, XRD data demonstrates no bands are detected for Ag or Ag_2_O in the Ag@Mg_4_Ta_2_O_9_ nanoparticles. The occurrence of O^2−^, Ta^5+^, Mg^2+^ ions and silver metal were clearly confirmed using XPS spectra for 2.0 wt.% Ag@ Mg_4_Ta_2_O_9_ nanocomposite. Moreover, the TEM images show the size of the Mg_4_Ta_2_O_9_ effect with adding of Ag as also provides from XRD data. However, the photocatalytic activity increases from 1 to 100% with increasing the silver content from 0 to 2.0 wt%. Carbon dioxide, NO_3_^−^, H_2_O, and Cl^−^ were produced after completing degradation of atrazine using the presence of this novel 2.0 wt% Ag@Mg_4_Ta_2_O_9_ nanoparticle.

## References

[1] Naghashkar NJ, El-Din MG (2007). Degradation of Aqueous Pharmaceuticals by Ozonation and Advanced Oxidation Processes: A Review. Sci. & Eng..

[2] Ternes TA (1998). Occurrence of drugs in German sewage treatment plants and rivers. Water Res..

[3] Arcand-Hoy LD, Nimrod AC, Benson WH (1998). Endocrine-Modulating Substances in the Environment: Estrogenic Effects of Pharmaceutical Products. Int. J Toxicolo..

[4] Kummerer K (2004). Resistance in the environment. J Antimicrob. Chemothera..

[5] Alinsafi A (2007). Treatment of textile industry wastewater by supported photocatalysis. Dyes Pigm..

[6] Eriksson E, Baun A, Mikkelsen PS, Ledin A (2007). Risk assessment of xenobiotics in stormwater discharged to Harrestrup Å, Denmark. Desalination..

[7] Neumann M (2002). The significance of entry routes as point and non-point sources of pesticides in small streams. Water Res..

[8] Jacobsen CS, Hjelmsø MH (2014). Agricultural soils, pesticides and microbial diversity. Curr. Opin. Biotechnol..

[9] Dong X (2009). Effects of atrazine on cytochrome P450 enzymes of zebrafish (Danio rerio). Chemosphere.

[10] Whyatt RM (2007). Within- and Between-Home Variability in Indoor-Air Insecticide Levels during Pregnancy among an Inner-City Cohort from New York City. Environ. Health Perspect..

[11] Miller SM, Sweet CW, DePinto JV, Hornbuckle KC (2000). Atrazine and Nutrients in Precipitation:  Results from the Lake Michigan Mass Balance Study. Environ. Sci. Technol..

[12] Banks KE, Hunter DH, Wachal DJ (2005). Hlorpyrifos in surface waters before and after a federally mandated ban. Environ. Int..

[13] Kaushik A, Sharma HR, Jain S, Dawra J, Kaushik CP (2010). Pesticide pollution of river Ghaggar in Haryana, India. Environ. Monit. Assess..

[14] Malato S (2002). Photocatalytic treatment of water-soluble pesticides by photo-Fenton and TiO_2_ using solar energy. Catal. Today.

[15] Roy SC, Varghese OK, Paulose M, Grimes CA (2010). Toward Solar Fuels: Photocatalytic Conversion of Carbon Dioxide to Hydrocarbons. ACS Nano..

[16] Atar N, Olgun A, Çolak F (2008). Thermodynamic, Equilibrium and Kinetic Study of the Biosorption of Basic Blue 41 using Bacillus maceran. Eng. Life Sci..

[17] Gupta VK, Mohan D, Sharma S, Sharma M (2000). Removal of Basic Dyes (Rhodamine B and Methylene Blue) from Aqueous Solutions Using Bagasse Fly Ash. Sep. Sci. Technol..

[18] Mohan N, Balasubramanian N, Subramanian V (2001). Electrochemical Treatment of Simulated Textile Effluent. Chem. Eng. Technol..

[19] Sojka-Ledakowicz J (2010). Application of membrane processes in closing of water cycle in a textile dye-house. Desalination..

[20] Velmurugan R, Swaminathan M (2011). An efficient nanostructured ZnO for dye sensitized degradation of Reactive Red 120 dye under solar light. Sol. Energy Mater. Sol. Cells.

[21] Beydoun D, Amal R, Low G, McEvoy S (1999). Role of Nanoparticles in Photocatalysis. J. Nanopart. Res..

[22] Esfandyarpour R, Esfandyarpour H, Javanmard M, Harris JS, Davis RW (2013). Label-free electronic probing of nucleic acids and proteins at the nanoscale using the nanoneedle biosensor. Biomicrofluidics..

[23] Esfandyarpour R, Esfandyarpour H, Harris JS, Davis RW (2013). Simulation and fabrication of a new novel 3D injectable biosensor for high throughput genomics and proteomics in a lab-on-a-chip device. Nanotech..

[24] Esfandyarpour R, Esfandyarpour H, Javanmard M, Harris JS, Davis RW (2013). Microneedle biosensor: A method for direct label-free real time protein detection. Sens. Actuators B..

[25] Pekakis PA, Xekoukoulotakis NP, Mantzavinos D (2006). Treatment of textile dyehouse wastewater by TiO_2_ photocatalysis. Water Res..

[26] Bessekhouad Y (2006). UV–vis versus visible degradation of Acid Orange II in a coupled CdS/TiO_2_ semiconductors suspension. J. Photochem. Photobiol. A.

[27] Pan L (2013). Enhancement of visible-light-induced photodegradation over hierarchical porous TiO_2_ by nonmetal doping and water-mediated dye sensitization. Appl. Surf. Sci..

[28] Dostanic J (2012). Photodegradation of an azo pyridone dye using TiO_2_ films prepared by the spray pyrolysis method. Chem. Eng. J..

[29] Kawahara T, Ozawa T, Iwasaki M, Tada H, Ito S (2003). Photocatalytic activity of rutile–anatase coupled TiO_2_ particles prepared by a dissolution–reprecipitation method. J. Colloid Interface Sci..

[30] Weng B, Lu K-Q, Tang Z, Ming Chen H, Xu Y-J (2018). Stabilizing ultrasmall Au clusters for enhanced photoredox catalysis. Nature Communications..

[31] Lu K-Q, Xin X, Zhang N, Tang Z-R, Xu Y-J (2018). Photoredox catalysis over graphene aerogelsupported Composites. J. Mater. Chem. A..

[32] Zhang N, Yang M-Q, Liu S, Sun Y, Xu Y-J (2015). Waltzing with the Versatile Platform of Graphene to Synthesize Composite Photocatalysts. Chem. Rev..

[33] Sakkas VA, Albanis TA (2003). Photocatalyzed degradation of the biocides chlorothalonil and dichlofluanid over aqueous TiO_2_ suspensions. Appl. Catal. B.

[34] Shi R, Wang Y, Li D, Xu J, Zhu Y (2010). Synthesis of ZnWO_4_ nanorods with [1 0 0] orientation and enhanced photocatalytic properties. Appl. Catal. B.

[35] Chen XP (2011). ZnWO_4_: Eu^3+^ nanorods: A potential tunable white light-emitting phosphors. J. Alloy. Compd..

[36] Sun DC, Senz S, Hesse D (2004). Topotaxial formation of Mg_4_Ta_2_O_9_ and MgTa_2_O_6_ thin films by vapour-solid reactions on MgO (001) crystals. J. Eur. Ceram. Soc..

[37] Ferrai CR, Hernandes AC (2002). MgTa_2_O_6_ and ZnTa_2_O_6_ ceramics from oxide precursors. J. Eur. Ceram. Soc..

[38] Thirumal M, Ganguli AK (2001). Synthesis and dielectric properties of magnesium niobate-magnesium tantalate solid solutions. Mater. Res. Bull..

[39] Kato H, Kudo A (1998). New tantalate photocatalysts for water decomposition into H_2_ and O_2_. Chem. Phys. Lett..

[40] Bernard J, Houivet D, Fallah JE, Haussonne JM (2004). MgTiO_3_ for Cu base metal multilayer ceramic capacitors. J. Eur. Ceram. Soc..

[41] Gaikwad AB, Navale SC, Samuel V, Murugan AV, Ravi V (2006). A co-precipitation technique to prepare BiNbO_4_, MgTiO_3_ and Mg_4_Ta_2_O_9_ powders. Mat. Res. Bull..

[42] Navale SC, Ravi V (2005). Preparation of fine MgTa_2_O_6_ and Mg_4_Ta_2_O_9_ powders by chemical methods. Mat. Sci. & Eng.: B.

[43] Kim J-S, Choi E-S, Ryu K-W, Bae S-G, Lee Y-H (2009). Microwave dielectric properties of Mg_4_Ta_2_O_9_ ceramics with TiO_2_ additions for dielectric resonator oscillator. Mat. Sci. & Eng.: B.

